# 12-Ethyl-6a,10a-di­hydro-5*H*-6-oxachrysene

**DOI:** 10.1107/S2414314620002862

**Published:** 2020-03-05

**Authors:** Alan J. Lough, Austin Pounder, William Tam

**Affiliations:** a Department of Chemistry, University of Toronto, Toronto, Ontario, M5S 3H6, Canada; b Department of Chemistry, University of Guelph, Guelph, Ontario, N1G 2W1, Canada; University of Aberdeen, Scotland

**Keywords:** crystal structure, regioisomer, fused-rings, weak hydrogen bonds

## Abstract

In the title compound, C_19_H_16_O, the pyran ring is in a half-chair conformation. The essentially planar naphthalene ring system (r.m.s. deviation = 0.020 Å) forms a dihedral angle of 14.37 (5)° with the fused benzene ring. In the crystal, pairs of mol­ecules are connected into inversion dimers by weak C—H⋯O hydrogen bonds to generate *R*_2_^2^(6) loops.

## Abstract 2

In the title compound, the pyran ring is in a half-chair conformation. The essentially planar naphthalene ring system forms a dihedral angle of 14.37 (5)° with the fused benzene ring. In the crystal, pairs of mol­ecules are connected into inversion dimers by weak C—H⋯O hydrogen bonds to generate 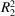 (6) loops.

## Graphical abstract

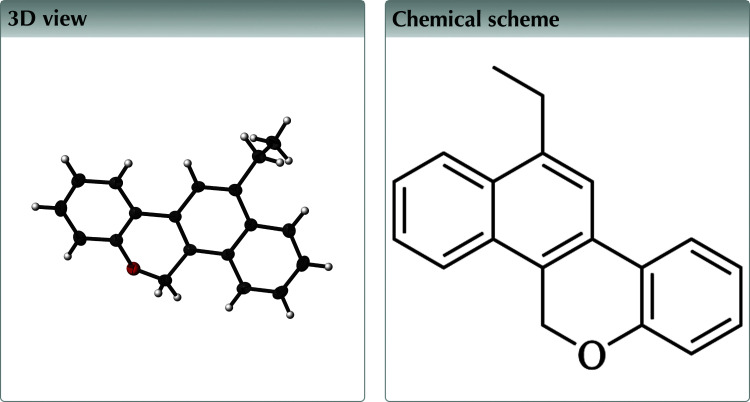

## Structure description

In past years, our research group has investigated the effects of various C_1_-substituted oxabenzonorbornadienes (OBD) on controlling the regioselectivity of ring-opening reactions (Hill *et al.*, 2019 ▸; Hill & Tam, 2019 ▸; Edmunds *et al.*, 2015 ▸; Raheem *et al.*, 2014 ▸; Boutin *et al.*, 2019 ▸). To date, there have been very limited investigations into the C_1_-tethered intra­molecular ring-opening reactions of OBD and derivatives thereof (Loh *et al.*, 2016 ▸; Wicks *et al.*, 2019 ▸). Recently, our group looked into the palladium-catalysed intra­molecular aryl­ation of oxabenzonorbornadiene derivatives. Reaction of the C_1_-tethered unsymmetrical OBD **I** (see Fig. 3 ▸) in the presence of Pd(PPh_3_)_2_Cl_2_, Zn powder, and Et_3_N in aceto­nitrile afforded the dehydrated **II** and hydrated **III** ring-opened products in an 87% and 3% yield respectively. Of the two potential regioisomers, the reaction was found to give solely the C_2_-cyclized regioisomer.

**Figure 1:**
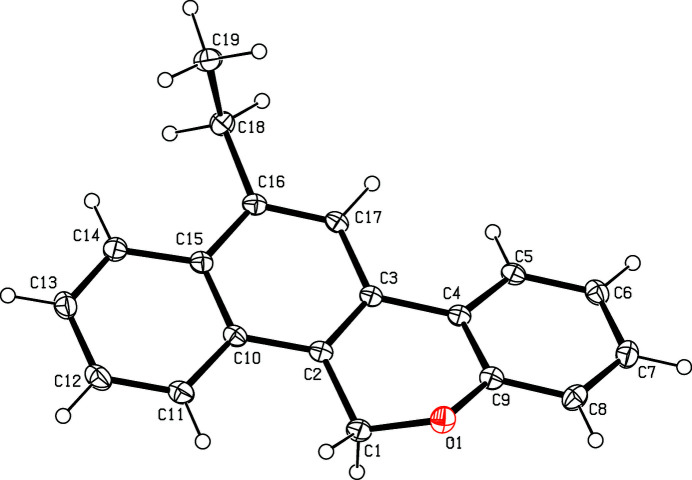
The mol­ecular structure of the title compound with displacement ellipsoids drawn at the 30% probability level.

**Figure 2:**
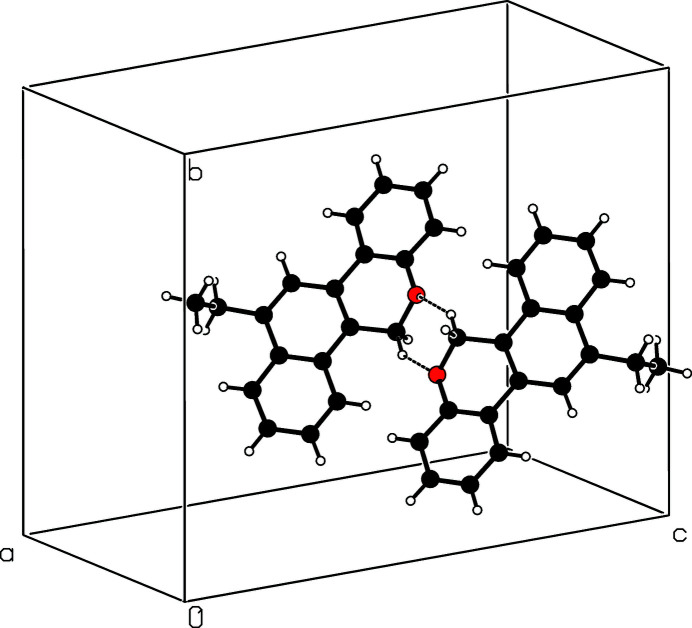
Part of the crystal structure with weak hydrogen bonds shown as dashed lines.

The mol­ecular structure of the title compound is shown in Fig. 1 ▸. The pyran ring (C1—C4/O1/C9) is in a half-chair conformation with atoms O1 and C1 displaced from the mean plane of the other four atoms by −0.260 (1) and 0.330 (1) Å, respectively. The essentially planar naphthalene ring system (C2/C3/C10–C17) forms a dihedral angle of 14.37 (5)° with the fused benzene ring (C4–C9). In the crystal, pairs of mol­ecules are connected into inversion dimers (Fig. 2 ▸) by weak C—H⋯O hydrogen bonds (Table 1 ▸) to generate 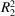 (6) loops.

## Synthesis and crystallization

**Figure 3:**
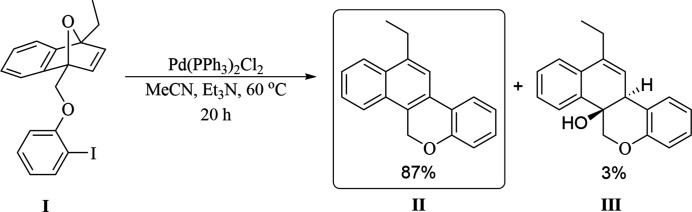
The reaction scheme.

To a 2 dram vial was added the aryl iodide-tethered oxabenzonorbornadiene **I** (73.0 mg, 0.180 mmol) (see Fig. 3 ▸). The vial was purged with argon for 5 minutes before being imported into an inert glove box. Inside the glove box, Pd(PPh_3_)_2_Cl_2_ (10.8 mg, 15.3 µmol, 8 mol%) and Zn powder (41.3 mg, 0.631 mmol) were added to the vial and dissolved in 1 ml of MeCN, followed by subsequent addition of Et_3_N (0.12 ml, 8 mol%). The vial was exported and stirred at 333 K for 20 h. The crude solution was purified by flash chromatography (EtOAc:hexa­nes, 15: 85) by loading directly onto the column to obtained ring-opened products **II** (23.5 mg, 0.0904 mmol, 87%) and **III** (1.5 mg, 5.34 µmol, 3%) as white solids. These were subsequently crystallized from DCM solution by slow evaporation of the solvent to afford product **II** as colourless crystals.

## Refinement

Crystal data, data collection and structure refinement details are summarized in Table 2 ▸.

## Supplementary Material

Crystal structure: contains datablock(s) I. DOI: 10.1107/S2414314620002862/hb4339sup1.cif

Structure factors: contains datablock(s) I. DOI: 10.1107/S2414314620002862/hb4339Isup2.hkl

Supporting information file. DOI: 10.1107/S2414314620002862/hb4339Isup3.cml

CCDC reference: 1987308

Additional supporting information:  crystallographic information; 3D view; checkCIF report

## Figures and Tables

**Table 1:** Hydrogen-bond geometry (Å, °)

*D*—H⋯*A*	*D*—H	H⋯*A*	*D*⋯*A*	*D*—H⋯*A*
C1—H1*A*⋯O1^i^	0.99	2.49	3.3514 (14)	146

Symmetry code: (i) 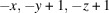 .

**Table 2:** Experimental details

Crystal data	
Chemical formula	C_19_H_16_O
*M*_r_	260.32
Crystal system, space group	Orthorhombic, *P* *b* *c* *a*
Temperature (K)	150
*a*, *b*, *c* (Å)	9.4802 (3), 14.9824 (7), 18.6565 (7)
*V* (Å^3^)	2649.90 (18)
*Z*	8
Radiation type	Mo *K*α
μ (mm^−1^)	0.08
Crystal size (mm)	0.34 × 0.28 × 0.20

Data collection	
Diffractometer	Bruker Kappa APEX DUO CCD
Absorption correction	Multi-scan (*SADABS*; Krause *et al.*, 2015 ▸)
*T*_min_, *T*_max_	0.712, 0.746
No. of measured, independent and observed [*I* > 2σ(*I*)] reflections	12018, 3052, 2504
*R*_int_	0.024
(sin θ/λ)_max_ (Å^−1^)	0.651

Refinement	
*R*[*F* ^2^ > 2σ(*F* ^2^)], *wR*(*F* ^2^), *S*	0.038, 0.101, 1.04
No. of reflections	3052
No. of parameters	182
H-atom treatment	H-atom parameters constrained
Δρ_max_, Δρ_min_ (e Å^−3^)	0.22, −0.20

Computer programs: *APEX3* and *SAINT* (Bruker, 2018 ▸), *SHELXT2014/5* (Sheldrick, 2015*a* ▸), *SHELXL2018/3* (Sheldrick, 2015*b* ▸), *PLATON* (Spek, 2020 ▸) and *publCIF* (Westrip, 2010 ▸).

## full crystallographic data

### Crystal data

**Table:**

C_19_H_16_O	*D*_x_ = 1.305 Mg m^−^^3^
*M**_r_* = 260.32	Mo *K*α radiation, λ = 0.71073 Å
Orthorhombic, *P**b**c**a*	Cell parameters from 4405 reflections
*a* = 9.4802 (3) Å	θ = 2.7–27.5°
*b* = 14.9824 (7) Å	µ = 0.08 mm^−^^1^
*c* = 18.6565 (7) Å	*T* = 150 K
*V* = 2649.90 (18) Å^3^	Block, colourless
*Z* = 8	0.34 × 0.28 × 0.20 mm
*F*(000) = 1104	

### Data collection

**Table:**

Bruker Kappa APEX DUO CCD diffractometer	2504 reflections with *I* > 2σ(*I*)
Radiation source: sealed tube with Bruker Triumph monochromator	*R*_int_ = 0.024
φ and ω scans	θ_max_ = 27.6°, θ_min_ = 2.2°
Absorption correction: multi-scan (SADABS; Krause *et al.*, 2015)	*h* = −12→7
*T*_min_ = 0.712, *T*_max_ = 0.746	*k* = −14→19
12018 measured reflections	*l* = −23→17
3052 independent reflections	

### Refinement

**Table:**

Refinement on *F*^2^	Primary atom site location: structure-invariant direct methods
Least-squares matrix: full	Hydrogen site location: inferred from neighbouring sites
*R*[*F*^2^ > 2σ(*F*^2^)] = 0.038	H-atom parameters constrained
*wR*(*F*^2^) = 0.101	*w* = 1/[σ^2^(*F*_o_^2^) + (0.045*P*)^2^ + 0.903*P*] where *P* = (*F*_o_^2^ + 2*F*_c_^2^)/3
*S* = 1.04	(Δ/σ)_max_ < 0.001
3052 reflections	Δρ_max_ = 0.22 e Å^−^^3^
182 parameters	Δρ_min_ = −0.20 e Å^−^^3^
0 restraints	

### Special details

**Table:**

Geometry. All esds (except the esd in the dihedral angle between two l.s. planes) are estimated using the full covariance matrix. The cell esds are taken into account individually in the estimation of esds in distances, angles and torsion angles; correlations between esds in cell parameters are only used when they are defined by crystal symmetry. An approximate (isotropic) treatment of cell esds is used for estimating esds involving l.s. planes.

### Fractional atomic coordinates and isotropic or equivalent isotropic displacement parameters (Å^2^)

**Table:**

	*x*	*y*	*z*	*U*_iso_*/*U*_eq_	
O1	0.14745 (9)	0.56200 (6)	0.52772 (5)	0.0288 (2)	
C1	0.19567 (12)	0.47662 (8)	0.50242 (7)	0.0253 (3)	
H1A	0.113259	0.440513	0.487550	0.030*	
H1B	0.243243	0.444816	0.542161	0.030*	
C2	0.29601 (11)	0.48465 (7)	0.44035 (6)	0.0203 (2)	
C3	0.38550 (11)	0.55736 (7)	0.43874 (6)	0.0195 (2)	
C4	0.37337 (12)	0.62461 (7)	0.49599 (6)	0.0205 (2)	
C5	0.47302 (12)	0.69135 (8)	0.50892 (6)	0.0233 (2)	
H5A	0.557021	0.692806	0.481172	0.028*	
C6	0.45198 (13)	0.75556 (8)	0.56139 (7)	0.0268 (3)	
H6A	0.520398	0.800926	0.568953	0.032*	
C7	0.33054 (14)	0.75316 (9)	0.60274 (7)	0.0296 (3)	
H7A	0.315739	0.797104	0.638675	0.036*	
C8	0.23061 (13)	0.68699 (9)	0.59193 (7)	0.0289 (3)	
H8A	0.147770	0.685153	0.620547	0.035*	
C9	0.25251 (12)	0.62354 (8)	0.53905 (6)	0.0233 (3)	
C10	0.30578 (12)	0.41727 (7)	0.38677 (6)	0.0213 (2)	
C11	0.21812 (13)	0.34026 (8)	0.38753 (7)	0.0271 (3)	
H11A	0.148683	0.334068	0.423906	0.033*	
C12	0.23206 (14)	0.27487 (8)	0.33676 (7)	0.0321 (3)	
H12A	0.172461	0.223924	0.338117	0.038*	
C13	0.33420 (15)	0.28297 (8)	0.28273 (7)	0.0318 (3)	
H13A	0.343672	0.237217	0.247801	0.038*	
C14	0.42019 (14)	0.35610 (8)	0.27990 (6)	0.0268 (3)	
H14A	0.488670	0.360520	0.242901	0.032*	
C15	0.40889 (12)	0.42557 (7)	0.33134 (6)	0.0216 (2)	
C16	0.49793 (12)	0.50263 (8)	0.32932 (6)	0.0218 (2)	
C17	0.48407 (12)	0.56564 (7)	0.38189 (6)	0.0212 (2)	
H17A	0.542758	0.616993	0.380285	0.025*	
C18	0.60196 (13)	0.51671 (8)	0.26905 (7)	0.0284 (3)	
H18A	0.651154	0.459737	0.259180	0.034*	
H18B	0.673658	0.560771	0.284435	0.034*	
C19	0.53232 (16)	0.54966 (9)	0.20008 (7)	0.0357 (3)	
H19A	0.604987	0.561189	0.163888	0.054*	
H19B	0.480278	0.604847	0.209855	0.054*	
H19C	0.467017	0.504057	0.182266	0.054*	

### Atomic displacement parameters (Å^2^)

**Table:**

	*U*^11^	*U*^22^	*U*^33^	*U*^12^	*U*^13^	*U*^23^
O1	0.0198 (4)	0.0327 (5)	0.0339 (5)	−0.0022 (3)	0.0041 (4)	−0.0043 (4)
C1	0.0218 (5)	0.0262 (6)	0.0279 (6)	−0.0031 (5)	0.0016 (5)	0.0020 (5)
C2	0.0166 (5)	0.0218 (5)	0.0224 (6)	0.0010 (4)	−0.0030 (4)	0.0044 (4)
C3	0.0172 (5)	0.0201 (5)	0.0213 (5)	0.0022 (4)	−0.0026 (4)	0.0034 (4)
C4	0.0199 (5)	0.0209 (5)	0.0206 (5)	0.0027 (4)	−0.0034 (4)	0.0038 (4)
C5	0.0231 (5)	0.0223 (6)	0.0245 (6)	0.0005 (4)	−0.0022 (5)	0.0043 (5)
C6	0.0307 (6)	0.0223 (6)	0.0273 (6)	0.0001 (5)	−0.0077 (5)	0.0019 (5)
C7	0.0367 (7)	0.0272 (6)	0.0250 (6)	0.0068 (5)	−0.0047 (5)	−0.0033 (5)
C8	0.0270 (6)	0.0341 (7)	0.0255 (6)	0.0051 (5)	0.0018 (5)	−0.0013 (5)
C9	0.0214 (5)	0.0246 (6)	0.0240 (6)	0.0008 (4)	−0.0031 (4)	0.0025 (5)
C10	0.0207 (5)	0.0207 (5)	0.0224 (6)	−0.0001 (4)	−0.0054 (4)	0.0042 (4)
C11	0.0283 (6)	0.0260 (6)	0.0270 (6)	−0.0049 (5)	−0.0048 (5)	0.0042 (5)
C12	0.0392 (7)	0.0244 (6)	0.0325 (7)	−0.0081 (5)	−0.0100 (6)	0.0036 (5)
C13	0.0452 (8)	0.0230 (6)	0.0270 (6)	0.0013 (5)	−0.0084 (6)	−0.0017 (5)
C14	0.0329 (6)	0.0249 (6)	0.0227 (6)	0.0046 (5)	−0.0023 (5)	0.0022 (5)
C15	0.0228 (5)	0.0210 (5)	0.0209 (6)	0.0039 (4)	−0.0045 (4)	0.0037 (4)
C16	0.0190 (5)	0.0230 (5)	0.0235 (6)	0.0030 (4)	−0.0012 (4)	0.0048 (4)
C17	0.0182 (5)	0.0195 (5)	0.0260 (6)	−0.0008 (4)	−0.0011 (4)	0.0035 (4)
C18	0.0259 (6)	0.0275 (6)	0.0317 (7)	0.0020 (5)	0.0072 (5)	−0.0001 (5)
C19	0.0441 (8)	0.0337 (7)	0.0292 (7)	0.0033 (6)	0.0094 (6)	0.0065 (5)

### Geometric parameters (Å, º)

**Table:**

O1—C9	1.3736 (14)	C10—C15	1.4284 (16)
O1—C1	1.4381 (15)	C11—C12	1.3691 (18)
C1—C2	1.5034 (16)	C11—H11A	0.9500
C1—H1A	0.9900	C12—C13	1.403 (2)
C1—H1B	0.9900	C12—H12A	0.9500
C2—C3	1.3810 (15)	C13—C14	1.3666 (18)
C2—C10	1.4237 (16)	C13—H13A	0.9500
C3—C17	1.4189 (16)	C14—C15	1.4198 (16)
C3—C4	1.4728 (16)	C14—H14A	0.9500
C4—C5	1.3967 (16)	C15—C16	1.4307 (16)
C4—C9	1.3995 (16)	C16—C17	1.3676 (16)
C5—C6	1.3869 (17)	C16—C18	1.5105 (16)
C5—H5A	0.9500	C17—H17A	0.9500
C6—C7	1.3864 (18)	C18—C19	1.5281 (18)
C6—H6A	0.9500	C18—H18A	0.9900
C7—C8	1.3860 (18)	C18—H18B	0.9900
C7—H7A	0.9500	C19—H19A	0.9800
C8—C9	1.3857 (17)	C19—H19B	0.9800
C8—H8A	0.9500	C19—H19C	0.9800
C10—C11	1.4221 (16)		
			
C9—O1—C1	114.65 (9)	C12—C11—C10	121.15 (12)
O1—C1—C2	112.50 (9)	C12—C11—H11A	119.4
O1—C1—H1A	109.1	C10—C11—H11A	119.4
C2—C1—H1A	109.1	C11—C12—C13	120.12 (12)
O1—C1—H1B	109.1	C11—C12—H12A	119.9
C2—C1—H1B	109.1	C13—C12—H12A	119.9
H1A—C1—H1B	107.8	C14—C13—C12	120.58 (12)
C3—C2—C10	120.27 (10)	C14—C13—H13A	119.7
C3—C2—C1	117.94 (10)	C12—C13—H13A	119.7
C10—C2—C1	121.69 (10)	C13—C14—C15	121.11 (12)
C2—C3—C17	119.33 (10)	C13—C14—H14A	119.4
C2—C3—C4	118.42 (10)	C15—C14—H14A	119.4
C17—C3—C4	122.25 (10)	C14—C15—C10	118.50 (11)
C5—C4—C9	117.57 (11)	C14—C15—C16	121.96 (11)
C5—C4—C3	124.21 (10)	C10—C15—C16	119.55 (10)
C9—C4—C3	118.19 (10)	C17—C16—C15	118.77 (10)
C6—C5—C4	121.42 (11)	C17—C16—C18	120.02 (10)
C6—C5—H5A	119.3	C15—C16—C18	121.17 (10)
C4—C5—H5A	119.3	C16—C17—C3	122.62 (10)
C7—C6—C5	119.62 (11)	C16—C17—H17A	118.7
C7—C6—H6A	120.2	C3—C17—H17A	118.7
C5—C6—H6A	120.2	C16—C18—C19	112.94 (10)
C8—C7—C6	120.35 (12)	C16—C18—H18A	109.0
C8—C7—H7A	119.8	C19—C18—H18A	109.0
C6—C7—H7A	119.8	C16—C18—H18B	109.0
C9—C8—C7	119.46 (12)	C19—C18—H18B	109.0
C9—C8—H8A	120.3	H18A—C18—H18B	107.8
C7—C8—H8A	120.3	C18—C19—H19A	109.5
O1—C9—C8	117.48 (11)	C18—C19—H19B	109.5
O1—C9—C4	120.86 (11)	H19A—C19—H19B	109.5
C8—C9—C4	121.57 (11)	C18—C19—H19C	109.5
C11—C10—C2	122.03 (11)	H19A—C19—H19C	109.5
C11—C10—C15	118.54 (11)	H19B—C19—H19C	109.5
C2—C10—C15	119.41 (10)		
			
C9—O1—C1—C2	−48.75 (13)	C1—C2—C10—C11	2.20 (17)
O1—C1—C2—C3	33.77 (14)	C3—C2—C10—C15	−0.03 (16)
O1—C1—C2—C10	−150.00 (10)	C1—C2—C10—C15	−176.18 (10)
C10—C2—C3—C17	2.03 (16)	C2—C10—C11—C12	−177.98 (11)
C1—C2—C3—C17	178.33 (10)	C15—C10—C11—C12	0.41 (17)
C10—C2—C3—C4	−178.90 (10)	C10—C11—C12—C13	0.06 (19)
C1—C2—C3—C4	−2.61 (15)	C11—C12—C13—C14	−0.33 (19)
C2—C3—C4—C5	167.10 (11)	C12—C13—C14—C15	0.10 (19)
C17—C3—C4—C5	−13.87 (17)	C13—C14—C15—C10	0.38 (17)
C2—C3—C4—C9	−14.86 (15)	C13—C14—C15—C16	179.98 (11)
C17—C3—C4—C9	164.17 (10)	C11—C10—C15—C14	−0.62 (15)
C9—C4—C5—C6	−1.40 (17)	C2—C10—C15—C14	177.81 (10)
C3—C4—C5—C6	176.66 (10)	C11—C10—C15—C16	179.77 (10)
C4—C5—C6—C7	0.86 (18)	C2—C10—C15—C16	−1.80 (15)
C5—C6—C7—C8	0.10 (18)	C14—C15—C16—C17	−178.02 (11)
C6—C7—C8—C9	−0.48 (19)	C10—C15—C16—C17	1.57 (16)
C1—O1—C9—C8	−150.11 (11)	C14—C15—C16—C18	4.26 (16)
C1—O1—C9—C4	33.24 (15)	C10—C15—C16—C18	−176.14 (10)
C7—C8—C9—O1	−176.72 (11)	C15—C16—C17—C3	0.47 (17)
C7—C8—C9—C4	−0.09 (18)	C18—C16—C17—C3	178.21 (10)
C5—C4—C9—O1	177.52 (10)	C2—C3—C17—C16	−2.31 (17)
C3—C4—C9—O1	−0.65 (16)	C4—C3—C17—C16	178.67 (10)
C5—C4—C9—C8	1.01 (17)	C17—C16—C18—C19	−100.43 (13)
C3—C4—C9—C8	−177.16 (10)	C15—C16—C18—C19	77.25 (14)
C3—C2—C10—C11	178.35 (10)		

### Hydrogen-bond geometry (Å, º)

**Table:**

*D*—H···*A*	*D*—H	H···*A*	*D*···*A*	*D*—H···*A*
C1—H1*A*···O1^i^	0.99	2.49	3.3514 (14)	146

Symmetry code: (i) −*x*, −*y*+1, −*z*+1.
